# Supplementary material for: Are carbon emissions trading and green financial instruments synergistic? -Comprehensive quantitative research based on content analysis
Source: PLoS One. 2024 Mar 7;19(3):e0298601. doi: 10.1371/journal.pone.0298601 (PMC10919601; doi:10.1371/journal.pone.0298601)
Supplement: S1 Data — (ZIP) [file pone.0298601.s001.zip › Data/地级市Z_F工作报告.zip!/╝¬┴╓/│ñ┤║╩╨/└┤╫╘╣1⁄2╓┌║┼í╛┬φ┐╦╩2╛▌═°í┐_2014.docx]

各位代表：

现在，我代表市人民政府，向大会报告工作，请各位代表审议，并请市政协委员提出意见。

一、2013年工作回顾

今年是长春发展史上极不寻常的一年。面对错综复杂的外部环境和接踵而至的困难挑战，面对加快发展和改善民生的繁重任务，在市委的坚强领导下，在市人大、市政协的监督支持下，我们紧紧团结和依靠全市人民，顽强拼搏、砥砺奋进，全面推进改革开放和现代化建设，经济社会发展取得新的可喜成绩。

——经济运行稳中有进、质量提升。预计地区生产总值超过5000亿元，增长9%，全口径财政收入增长14.8%，首次突破千亿元大关。

——民生福祉显著增进、惠及城乡。幸福长春行动计划全面实施，又为群众办了一批好事、实事。城镇居民人均可支配收入增长12.1%，农民人均纯收入增长13%，城乡居民储蓄存款余额增长13.6%，这些体现群众收入水平的指标均高于经济增速、上年增速，发展成果更多地为人民群众所共享。

——各项事业纵深推进、成效显著。改革开放不断深化，城市建设实现突破，社会事业全面进步，生态文明建设稳步实施，社会大局继续保持和谐稳定。

各位代表，在今年这样极其特殊的形势下，我们为巩固和发展全市稳增长、惠民生、促和谐的生动局面，突出狠抓了以下七个方面的工作：

（一）统筹三次产业，全力稳增长、调结构、增效益迈出了新步伐

坚持调整优化经济结构、提升发展质量效益，狠抓三次产业融合发展、互促共进，不仅稳定了当期增长，也为长远发展奠定了基础。

工业支撑有力。全面加大对重点企业扶持力度，全年产值超亿元企业达到639户，产值超10亿元企业达到66户。实施投资3000万元以上工业项目1250个，大众发动机、轿股变速箱、丰越RAV4、百克疫苗、富士康新材料、新力LED光源等一批项目建成投产，产业结构不断调整优化。大力推进科技创新，新增高新技术企业31户，省级以上企业技术中心达到96户，转化重大科技成果130余项，突破共性关键技术300余项。500余种新产品实现规模化生产，新产品产值率达到47%；工业企业实现利润780亿元，增长18%；工业增加值率提高1个百分点，万元增加值能耗降低4%。工业增长质量和效益稳步提升，对全市经济稳增长起到了决定性的支撑作用。

服务业繁荣活跃。出台并落实了促进服务业转型升级的若干政策，服务业增加值突破2000亿元。商贸流通体系日益完善，社会消费品零售总额增长12%。旅游业总收入685亿元，增长25%。房地产业健康发展，施工面积增长39.6%。引进一批区域性总部、研发中心，软件、服务外包、电子商务等高端服务业迅速成长，医疗、教育、会展、购物等区域性服务能力进一步强化。长春金融总部基地启动建设，汇丰银行落户长春，吉林股权交易所挂牌营业，富奥股份上市，小额贷款公司超过100家。服务业与工业契合度进一步加深，为促进全市经济稳增长发挥了不可替代的拉动作用。

农业转型步伐加快。实施农作物高光效栽培示范49.3万亩，推广玉米保护性耕作158万亩，建设万亩高产示范片区120个。大型农业机械数量增长41.5%，玉米、水稻机收率分别提高5.5和6个百分点。农业大户、专业合作社、家庭农场快速增加，土地流转面积比例达到21%，规模经营水平稳步提高。战胜春季低温内涝、夏季寡照多雨等不利因素，粮食产量再创历史新高。新增科学储粮仓5万套。加快实施现代畜牧业提升计划，新建标准化养殖小区204个，畜牧业产值增长8.4％。农业基础地位得到进一步巩固加强。

（二）强化项目拉动增量带动，城区、开发区、县域“三大板块”取得了新发展

坚持扩大增量、优化总量，依托“三大板块”，狠抓招商引资和项目建设，为长春加快发展打牢基础、积蓄后劲。全市实际利用内资、外资均增长20%以上，投资超亿元、超10亿元的项目均增加50%以上，全社会固定资产投资增长21%。

城区、开发区、县域齐头并进、竞相发展的态势日趋明显。城区成为服务业的主战场,投资超亿元服务业项目达到730余个，服务业税收占城区财政收入比例达65%以上。开发区成为产业集聚的主力军,市直五个开发区为全市贡献了70%的工业产值、60%的财政收入，是全市发展当之无愧的中坚力量。县域成为产城融合的主阵地,县城和重点乡镇建设不断加快，完成工业投资680亿元，财政收入突破100亿元。

“三大板块”相互促进、协调发展的局面初步形成。汽开区与农安合作启动建设烧锅零部件合作区，高新区与德惠合作的长德新区、经开区与九台合作的经九合作区稳步发展，正在形成国家级开发区带动县域发展的新格局。农安合隆、德惠米沙子、九台卡伦等县域开发区项目建设不断提速，正在与主城区融为一体。莲花山度假区迅速崛起，二道物流、九台空港建设全面铺开，长吉一体化进程不断加快。

（三）坚持深化改革开拓创新，发展活力、动力和潜力实现了新提升

坚持以改革添活力、增动力，狠抓重点领域和关键环节的改革攻坚，为全市发展提供了坚强保障和有力支撑。

做好“减法”，增强活力。继续深化行政审批制度改革，取消下放行政许可及审批项目123项，审批规定时限进一步压缩。积极开展交通运输、文化创意、鉴证咨询、研发服务等重点行业营业税改增值税试点，为企业税负降低、经济结构调整、发展方式转变提供制度支持，最大限度地激发市场活力。

做好“加法”，注入动力。大力发展民营经济，出台鼓励发展民营经济50条政策，为小微企业和个体工商户减免税收13.6亿元，全市注册登记私营企业、个体工商户数量分别增长12.3%、24.7%，民营经济主营业务收入增长16%。兴隆综合保税区通过国家考核验收、正式封关运营，长春拥有了直通世界的对外开放平台。

做好“乘法”，激发潜力。企业注册登记实行“非禁即入”、“先照后证”，九台国家级农村金融体制机制改革实验区启动实施，农村集体土地确权登记发证基本完成，集体林权制度配套改革全面启动，城建投融资体制改革扎实推进，这些关键性环节的破冰改革，必将极大地释放发展潜力，形成具有乘数作用的放大效应。

（四）奋力克难攻坚重点推进，城市建设、改造和管理取得了新突破

坚持以新型城镇化为引领，狠抓城市建设改造和市容市貌管理，实现了突破性的重大进展,城市承载能力和整体形象明显提升。

道路建设强力推进。“两横三纵”快速路克服了工期紧、任务重、投资高、难点多、压力大等诸多不利因素，在全市人民的支持下，实现了主线贯通、简易通车，改写了城市道路交通史，也标志着长春正式进入立体交通时代。昼夜兼程地完成了236条主次干道的大中修。地铁1、2号线建设顺利推进。54路有轨既有线路改造竣工通车，西延长线实现贯通。

公用设施不断完善。3座净水厂改续建工程进展顺利，城区西部供水紧张状况明显缓解。新增供热能力1550万平方米。改造供热、供水地下管网490公里。加快建设6座污水处理厂，伊通河城区段基本完成污水截留。新建续建百花园等公园14个，新建绿地39块，新植街路40条，新增城市绿地600公顷。

城市环境持续改善。强化空气污染综合整治，启动天然气替代燃煤供热试点，撤并改造分散采暖锅炉164座，淘汰治理黄标车5500台，开展道路降尘除尘作业，推行大田秸秆禁烧政策。持续深入地开展市容环境整治攻坚，尽最大努力消除城市建设改造对市容环境的影响和冲击。

规划管理明显加强。认真落实城市总体规划、土地利用总体规划，规划和土地管理水平进一步提高。科学调度城建重点工程施工时序，从严整治机动车非法营运等管理顽疾，全面加大交通组织疏导力度，确保城市交通在极端困难情况下能够维持正常运转。

特别需要指出的是，在今年城市管理和交通拥堵面临前所未有的压力下，全市人民给予了最大限度的包容和理解，更加坚定了我们建设好、管理好这座城市的信心和决心。

（五）致力建设幸福长春，全心全意为群众办实事、做好事、解难题收获了新成果

坚持把民生作为第一目标，今年全市75%的新增财力投入到民生领域，比上年提高了5个百分点。

就业水平稳步提高。积极扶持高校毕业生、农村转移劳动力、城镇困难就业人员就业，城镇登记失业率始终保持在4%以下。职工月最低工资标准增长14.8%，达到1320元。城镇居民人均可支配收入、农民人均纯收入分别达到25720元、10240元。

养老保障日益完善。养老保险扩面取得重大突破，职工参保率达到93.5%，城乡居民参保率达到90%，基本实现城乡养老保险全覆盖。企业退休人员养老金人均提高181元，实现“九连增”。进一步提高了养老机构建设与运营补贴标准，新增养老床位1200张。

大病保险初步建立。新农合、居民医保各有40种大病列入保障范围，最高报销比例分别达到80%和85%。城镇居民医保50种常规病种实行定额治疗，11种大病实现低自付治疗。居民、职工医保门诊报销限额分别提高50%、66.7%，新农合年度报销封顶线提高25%。

居住条件不断改善。扎实推进保障性住房建设，建成公租房6000套、廉租房4000套，综合整治老旧散小区36个，完成老旧楼宇“暖房子”改造1000万平方米。拆除棚户区91.8万平方米，建设回迁房1.3万套。房地产交易市场落成开业。

居民出行更加便捷。改造居民巷道1000余条。新建公交候车亭114座，更新增加公交车500台。新建农村公路1512.1公里，通屯率达到65%。扎实推进167个省级新农村示范村建设。绿化美化村屯384个。解决43.1万农村人口饮水安全问题。

食品安全得到强化。取缔无证无照食品生产经营者260户，查处食品药品违法违规案件8300余件。290个食品安全快检站（室）投入使用，对市场流通食品高密度抽检。药品质量监管可追溯体系初步建立，对药品批发企业实施电子监管。

各类教育均衡发展。新建改建公办幼儿园15所，新增幼教学位4000个。新建3所城乡结合部九年一贯制学校，标准化改造60所中小学校园设施。城区中小学生均公用经费上调200元。52个大学区建立教育资源共享机制，首批623名教师交流轮岗。建立了农民工子女入学“绿色通道”，1万余名学生受益。启动城区适龄未入学残障儿童送教上门试点。国家职业教育综合改革试点稳步推进。免除2.5万名农村及涉农专业中职学生学费。

健康服务深入实施。启动了第二轮“健康长春行动计划”，顺利通过国家基本公共卫生服务专项考核。受世界卫生组织邀请，长春在第八届全球健康促进大会上做经验介绍。5家市级公立医院实施基本药物制度，取消药品销售加成。免费实施孕前优生健康检查及乙肝病毒母婴传播阻断工程，出生人口素质进一步提高。

文化惠民扎实推进。国家公共文化服务体系示范区通过验收，县（区）、乡、村群众文化基础设施达到国家规定标准。历史文化遗产保护实现重大突破，伪满皇宫等8处18项不可移动文物被核定为全国重点文物保护单位。群众艺术馆、朝鲜族艺术馆开工建设，博物馆、规划展览馆完成主体工程。新建城市社区健身路径94套，为10个乡镇及100个行政村安装健身器材。“全国文明城市”创建工作不断取得新成果。

社会救助惠及城乡。开展了万户特困户结对救助活动。城乡最低生活保障标准、农村五保供养标准均提高7%以上。实行城乡低保及低保边缘家庭中小学生营养午餐补贴制度，惠及贫困学生2.3万人。城市、农村“三无一靠”成年重度残疾人生活补贴标准分别提高33%和50%。城区“三无”、农村五保等困难群体在定点医疗机构就医基本实现“先住院、后付费”。办理困难群众法律援助案件2900余件，基本做到“应援尽援”。

（六）深刻汲取惨痛教训，深入持续地查隐患、抓整治、保安全积累了新经验

坚持“生命至上、安全第一”，深刻汲取“6•3”特大火灾事故教训，痛定思痛、举一反三，全力狠抓了安全隐患大检查、大整改、大演练活动。

强化了责任落实。全面落实安全生产主体的第一责任，3.5万人员密集型单位开展应急安全演练，14.6万有固定经营场所企业建立安全隐患自查自报机制，企业负责人及安全管理人员普遍接受安全知识培训。

强化了能力建设。深入开展企业安全生产标准化建设，煤矿、非煤矿山、危险化学品三个高危行业安全设施、管理水平均达到三级以上标准。581栋城区D级危房居民全部迁出并予以过渡安置。改造高危燃气管网240公里，完成燃气入户安检81万户。

强化了安全监管。先后成立293个专家组、1.2万个督查组对全市安全生产责任单位进行地毯式排查，累计排查整改隐患6.3万项，治理消除重大隐患385项，关闭取缔企业358户，118户重大危险源单位全部落实监管措施。

强化了长效机制。制定出台并严格落实安全发展战略指导意见、强化企业安全生产主体责任、安全生产分类分级管理等一系列制度措施，努力从源头消除安全隐患，建立安全发展的长效机制。

强化了治安整治。深入开展社会治安综合整治，110刑事警情、治安警情、“两抢”案件数量分别下降17.45%、43.39%、42.2%，有重大影响案件全部告破，命案破案率保持在95%以上。新增治安高清监控探头2800余个。

强化了社会治理。加快幸福社区建设，全面推行“人性化、网格化、信息化”服务管理模式，城区社区用房均超过500平方米。村委会换届工作圆满完成。南关区、双阳区基层平安创建经验在全省推广。长春被授予全国社会管理综合治理最高荣誉“长安杯”。

（七）严格依法行政转变职能，政府转作风、强服务、提效能取得了新成效

坚持转变职能、转变作风。自觉接受人大及其常委会的法律监督、工作监督和政协的民主监督，坚持重大事项向人大报告、与政协协商制度。认真听取各民主党派、工商联以及无党派人士的意见建议。办理人大议案4件、人大代表建议226件、政协提案322件。

狠抓了“四风”整治。全面开展以“为民、务实、清廉”为主题的群众路线教育实践活动，深入查摆和整改形式主义、官僚主义、享乐主义和奢靡之风问题。从严落实中央“八项规定”，清理2543名吃空饷人员，取消67个公款出国团组，精简500余个会议，取消“吉OA”公安专段车号牌。政府作风建设取得了阶段性成效。

强化了效能建设。充分利用信访机构接待、市长公开电话、局长接待日、读报读网制度等各种渠道，掌握群众诉求、解决群众困难。认真组织“三满意”机关创建、“万人评议机关”活动，加大涉软案件查处力度，经济发展软环境进一步优化。强化依法治市，严格依法行政，坚持从严治政，法治政府建设取得新进展。深入开展“情系驻长官兵、共爱革命功臣”活动，双拥工作取得新成效。

国家安全、审计、质监、统计、民族、宗教、外事、侨务、供销、气象、地震、档案、保密、人防、地方志、红十字等工作都取得了新成绩。

各位代表，回顾一年来的工作，我们取得这样的成绩殊为不易。这是省委、省政府高度重视、亲切关怀的结果，是市委科学决策、正确领导的结果，是市人大、市政协有效监督、大力支持的结果，是全市上下同心同德、团结奋斗的结果。实践再一次雄辩地证明，只要我们始终在省委、省政府和市委的坚强领导下，紧紧团结依靠全市广大人民群众，任何艰难险阻都挡不住我们前进的脚步。在此，我代表市人民政府，向全市各族人民致以亲切的问候和崇高的敬意！向人大代表、政协委员，各民主党派、工商联和无党派人士、人民团体，向驻长部队指战员、武警官兵和中省直单位，向所有关心和支持长春的港澳台同胞、海外侨胞及国际友人表示衷心的感谢！

在肯定成绩的时候，我们也清醒地认识到，经济社会发展和政府工作中还存在许多问题和困难：经济总量偏小、结构不尽合理，调结构、转方式任务繁重而紧迫；科技成果转化率不高，科技创新对发展的支撑带动作用发挥得不够；资源环境压力明显增大，空气污染等环境问题开始显性化；机动车数量持续高速增长，道路需求与供给矛盾依然比较突出；安全基础设施相对薄弱，安全事故高发势头还没有从根本上得到遏制；城乡居民收入总体水平不高，部分群众生活还很困难；政府职能转变不够，廉政建设需要进一步加强。这些问题需要我们认真面对并着力加以解决。

二、2014年工作安排

明年是新一轮改革攻坚的起步之年，实施“十二五”规划的关键之年。纵观国内国际形势，经济下行、政策趋紧造成的外部发展环境依然复杂严峻，改善民生、维护稳定带来的内部发展压力仍然非常巨大，但我们有信心、有条件、有能力克服困难、应对挑战。我们的信心和力量来自市委对形势的科学判断和准确把握；来自已经制定并实施的应对挑战着眼长远的一系列政策措施；来自全面深化改革释放迸发的强大动力；来自工业化、城镇化快速推进催生的巨大需求；来自连续多年高强度投资积蓄的强劲发展势能；来自长春人民坚忍不拔、发奋图强的伟大力量。我们坚信，只要我们抓住机遇而不丧失机遇、奋力作为而不无所作为，就一定能够化危为机、克难而上，抢抓机遇、乘势快上，更好地战胜各种困难和挑战、更快地增强发展活力和动力，把长春全面振兴的大业不断推向新高度、实现新跨越！

明年政府工作的总体要求是: 深入学习贯彻落实党的十八届三中全会、省委十届三次全会和市委十二届四次全会精神，紧紧围绕主题主线，围绕建设幸福长春、率先全面建成小康社会目标，围绕科学发展、改善民生、建好城市、促进和谐工作大局，围绕城市转型升级新任务，坚持稳中求进工作总基调，把改革创新贯穿于经济社会发展各个领域各个环节，以改革创新促发展、促转型，努力推动经济建设、政治建设、文化建设、社会建设、生态文明建设取得新成效。

明年全市经济社会发展的主要预期目标是：地区生产总值增长10%，全口径财政收入增长11.5%，固定资产投资增长13%，规模以上工业增加值增长11%，服务业增加值增长10%，社会消费品零售总额增长10%，城镇居民人均可支配收入增长12%，农民人均纯收入增长13%，万元GDP能耗降低3%。

这里要着重说明，在社会主义市场经济条件下，政府提出地区生产总值增长速度目标，是一个预期性、指导性指标，是提出财政预算、社会就业、群众收入等经济指标的重要依据。由于国内外经济环境和市场变化影响，最终实现的增长速度与预期目标可能会有一定的差距。提出增长速度10%的目标，综合考虑了需要和可能等多种因素，既着眼于保持经济平稳较快发展，实现经济发展质量和效益得到提高又不会带来后遗症的速度，更注重引导各方面把主要精力和工作重点放到改革创新和转变发展方式上来，推动经济社会又好又快发展。为此，我们要抓好以下八项重点工作：

（一）以改革创新统领经济社会发展，加快推动城市转型升级

坚定不移地全面深化改革，积极破除一切妨碍科学发展的体制机制弊端，逐步把发展转到创新发展、统筹发展、绿色发展、开放发展、安全发展的轨道上来，努力打造长春经济社会发展的升级版。

遵循顶层设计抓落实。对于制定市场准入负面清单、建立城乡统一建设用地市场、完善地方税收体系、行政执法体制、收入分配调控体制机制等涉及面广、需要国家决策部署的改革，提前做好工作准备，积极开展政策对接，全面抓好贯彻落实，确保这些改革措施落到实处、取得成效。

围绕既定任务抓推进。对于基础教育、公立医院、文化管理体制、城建投融资体制、城市管理体制、国有资产管理体制、厂办大集体、社会组织管理体制、事业单位招聘制度、被征地农民养老保险制度、食品药品安全监管体制等方向明确、路径清晰的改革，要稳扎稳打、全力推进、尽快突破。

确定自选动作抓试点。对于创办地方性金融机构、产学研协同创新机制、不动产统一登记、城乡双向一体化、构建新型农业经营体系、农民财产权抵押担保转让、粮食收储仓储融资服务等需要深入摸索实践的改革，要试点先行、大胆探索、争取主动。

突出重点领域抓突破。全力抓好突出发展民营经济综合配套改革示范区试点，围绕市场准入、市场监管、公平竞争、金融服务等关键环节进行综合配套改革，使各项改革措施协同配合、良性互动、形成共振，为民营经济大发展快发展探索新途径、提供新动力。

（二）狠抓工业项目不动摇，依靠增量调整产业结构、转变发展方式

坚定不移地把发展工业的关键点敲定在工业项目上，以项目促转型、以项目增后劲。明年，工业投资计划安排1800亿元以上。不管遇到多大困难，都要坚决完成这项任务。

围绕龙头企业上项目。全力支持一汽、长客、大成等龙头企业强化自主创新、加快扩能步伐、集聚配套企业，壮大汽车、轨道客车、农产品加工支柱产业，加快建设三大世界级产业基地。

依托新兴产业上项目。谋划实施抗癌新药、高铁轮对、高温传感器、高氮合金、新能源客车、生物酶制剂、聚乳酸、LED大屏幕、商用飞机零部件等100个重点项目，加快发展医药健康、装备制造等优势产业，生物、光电信息、新能源、新能源汽车、新材料、节能环保等战略性新兴产业，尽快把生物、光电信息培育成新的优势产业。

凭借科技优势上项目。转化民用小卫星等100项关键核心技术，培育100个科技创新“小巨人”企业。进一步调整产品结构，提高产品附加值，工业增加值率提高1个百分点以上。

突出园区载体上项目。全力推动开发区转型升级，每个以工业为主的开发区都要提高投资强度、科技含量、投入产出比、集约发展水平，都要构建有品牌、有规模、有影响力的产业集群。

强力推进招商上项目。大力实施长吉图开发开放和长吉一体化战略，着力引进世界和国内500强企业，实施一批骨干支撑型重大项目，实际利用外资增长12%，利用内资增长15%。积极开拓国际市场，推动外贸出口稳步增长。兴隆综合保税区投入实质性运营。

（三）大力发展服务业，加快构建工业、服务业“双拉动”格局

坚定不移地促进服务业与其它产业深度融合，以服务业跨越提升，支撑工业转型升级。

加快提升生产性服务业。启动建设一汽物流产业园区，构建现代物流产业体系。抓好国家城市共同配送试点，降低城市物流成本。加快建设长春金融总部基地，力争一批金融企业入驻开业。积极发展中小型金融机构，促进融资担保机构规范发展。力争引进2家域外金融机构。企业上市要有新的更大突破。

改造提升传统服务业。加快建设南部新城核心区、长东北核心区、净月生态大街、西客站核心区、经开南区、汽车公园周边、莲花山度假区、铁北环铁商圈、吉林大路沿线、双阳奢岭等一批服务业聚集区。加强商贸流通体系建设，扩大城乡居民消费需求，培育新兴消费热点。保持房地产业健康发展态势。

积极培育新兴服务业。做大汽车服务、楼宇经济、服务外包产业，积极扶持数字动漫、影视传媒、民间工艺等文化产业，推动服务业全面扩容、提档升级。推动“智慧长春”建设，提升信息服务能力，促进信息消费。稳步发展会展经济。抓紧构建大旅游发展格局，旅游业总收入增长25%以上。

（四）突出以城带乡、以工促农，积极推动县域经济加快振兴、城乡一体化发展

坚定不移地加快构建新型城乡发展格局，抓住扩权强县的有利契机，全力推动县域经济突破跃升。

加快推进县域工业化。充分发挥国家级开发区的带动作用，加快建设长德新区、经九合作区、烧锅零部件合作区。推动农安合隆、德惠米沙子、九台卡伦加速与主城区融合，承接城市产业转移。四县（市）都要全力实施工业化，都要培育有特色的产业集群，确保工业投资增长15%以上。

加快推进县域城镇化。着力建设县城和一批重点乡镇，完善基础设施，发展社会事业，增强人口接纳能力，努力提高城镇化发展质量。推广莲花山度假区新型城镇化模式，逐步把符合条件的农业转移人口转为城镇居民，积极推进以人为核心的城镇化。大力提高城镇建成区人口密度，节约集约利用土地、能源、水等资源。

加快推进农业现代化。抓好16个现代农业示范区建设。集成推广保护性耕作、高光效栽培等技术措施，努力提高粮食综合生产能力和效益水平。农作物耕种收综合机械化水平提高2个百分点，土地流转面积比例提高3个百分点。加快“菜篮子”工程建设。继续实施现代畜牧业提升计划，新建扩建标准化养殖小区200个。继续实施三座大型灌区改造和德惠五大围堤除险加固。更新改造农防林600公顷。新建科学储粮仓2.5万套。

（五）坚持建设管理并重，全面加快绿色宜居森林城建设

坚定不移抓好城市建设管理，在继续抓好城市道路建设的基础上，把更多的精力转到旧城改造、生态建设和城市管理上来。重点实施“五大工程”：

实施路网改造建设工程。“两横三纵”快速路正式投入运行。加快建设地铁1、2号线。续建大众物流通道、解放物流通道、南部新城路网、机场快速路。启动建设吉林大路、南湖大路、河东路等延长线，拓宽改造皓月大路、西湖大路。打通一批卡脖路、断头路。

实施棚户区改造攻坚工程。力争用5年左右时间基本完成建成区内176处成片棚户区、209块零星（夹馅）棚户区及危倒房改造。这项任务的实施，不仅能够改善10.5万户居民居住条件，还可以从整体上提升城市面貌。

实施城市绿化美化工程。力争用3年时间通过国家森林城考核验收。年内高标准恢复“两横三纵”快速路周边绿地，提升伊通河城区段绿化水平，新建续建14个公园，新增城市绿地600公顷，植树造林3000公顷。

实施生产要素保障工程。五水厂竣工投入运行。建设天然气高压外环管网。新增供热能力1000万平方米。实施北郊污水处理、串湖污水处理新建扩建工程。进一步改善伊通河、饮马河等水系水质。继续推进生活垃圾焚烧发电厂、餐厨垃圾处理厂建设。

实施城市综合整治工程。全面实施新一轮的150天市容环境综合整治，努力实现城市管理水平的全面提升。坚持最严格的耕地保护制度，加强土地节约集约利用，严肃查处违法用地行为。

（六）纵深推进幸福长春行动计划，全力增进民生福祉改善人民生活

明年，我们要在强化政府基本公共服务的基础上，进一步加大民生投入，突出办好16件群众关心的难事、大事，不断提高城乡群众的幸福感和满意度。

实施就业富民。新增就业岗位12万个。对就业困难人员实行托底安置。零就业家庭至少一人就业。引导扶持1万人创业，推动创业成为富民的重要途径。努力增加低收入者收入，逐步扩大中等收入者比重，努力缩小城乡居民收入差距。

突出保障安民。稳步提高城乡养老保险参保率。解决6.3万名被征地农民养老保险问题。制定出台进一步发展养老服务业的优惠政策，力争年内新增养老床位4000张。城区低保家庭老、少、病、残人员低保补助标准上浮50%。建立支出型、急难型贫困家庭临时救助机制。

抓好安居乐民。继续实施“暖房子”工程，配套改造30个老旧散小区。新建廉租房600套、公租房1700套、棚户区回迁安置房1.8万套。通过市场化专业服务、业主自治相结合的方式解决物业弃管问题，尽快实现基本物业服务全覆盖。

突出环境助民。实施天然气替代燃煤工程，从严整治城市扬尘和工业污染排放，加快淘汰黄标车、燃煤小锅炉，可吸入颗粒物年均值下降10%左右。综合治理石头口门、新立城水库周边环境，确保水源地水质安全。开展农村环境卫生整治行动，167个村环境卫生设施达到省级新农村示范村标准。

开展服务便民。接收改造220座二次供水泵站。解决20万农村人口饮水安全问题。改造150公里高危燃气管网、100公里供热管网、100公里供水管网。更新一批公交车辆，科学设置公交线路，逐步提高公交智能化水平。新建一批过街天桥。启动建设一批立体停车场。

推动教育利民。采取以奖代补等多种方式，积极扶持面向大众、收费较低的普惠性民办幼儿园。创建30所新优质学校，100所公办中小学校园设施实现标准化，城区学校常规教学和信息技术装备全部达标，公办校教师合理交流轮岗比例争取达到10%，不断提高教育均衡发展水平。进一步健全家庭经济困难学生资助体系，不让一个孩子因贫失学。

强化医疗为民。居民医保大病保障病种由40种扩大到60种，低自付病种由11种扩大到21种。建立城乡医疗救助中心，把低保群众就医医药费自付比例降到10%以下。2/3以上的村设立公立卫生室，药品销售实现零差率。组织城乡医疗机构为468家养老机构实施对口医疗服务。

促进文化惠民。巩固扩大国家公共文化服务体系示范区创建成果，满足群众基本文化需求。图书馆、文化馆免费开放服务基本实现标准化、规范化。城区建设10条健身步道、安装50套健身路径。为 10个乡镇及100个行政村安装健身器材。群众艺术馆、朝鲜族艺术馆、博物馆建成竣工，规划展览馆投入使用。

（七）全面加强城市安全建设，创新社会治理长效机制和工作体系

坚定不移地树立“大安全”理念，围绕创建平安长春，全力推动安全发展。

全面强化责任监管。毫不松懈地开展安全隐患大检查、大整改、大演练活动，坚决做到减少一般事故、防范较大事故、遏制重大事故、杜绝特大事故。突出消防安全整治，强化公共消防设施建设，全力整改重大火灾隐患。

切实强化社会治理。扎实开展基层平安创建活动，健全完善社会治安综合治理体系。严厉打击各类刑事犯罪，坚决做到黄赌毒必治、黑恶势力必除，命案破案率保持在90%以上，进一步提高人民群众的安全感。

突出强化公共安全。严把驾驶员培训考试关口，从严整治机动车行车秩序。对住宅电梯实行分类管理，完成高层弃管电梯改造。加快地震监测网络建设，强化建设工程地震安全性管理，不断提高防灾减灾能力。

大力强化食品安全。深入创建食品安全城市，争取首批进入“国家食品安全示范城市”行列。推进食品药品监管信息化和技术支撑能力建设，尽快建成肉菜流通追溯体系，提升食品安全监管水平。

（八）加强政府自身建设，着力打造务实、高效、为民、廉洁政府

坚定不移地加强服务型政府建设，全面畅通“四大通道”，以政府职能的大转变，促进各项重点工作的大落实。

自觉接受监督，畅通“民主通道”。坚持向人大报告工作制度，认真落实人大及其常委会的决议、决定。积极支持政协履行政治协商、民主监督和参政议政职能，主动听取各民主党派、工商联、无党派人士意见。认真办理好人大代表议案、建议和政协提案，不断提高落实率。

简化行政审批，畅通“效能通道”。进一步削减地方行政审批事项，不符合现行法律规定、利用“红头文件”设定的管理、收费、罚款项目一律取消。创新行政监管方式，强化事中事后监管，认真负责地管好该管的事。把政府性债务纳入全口径预算管理，着力控制和化解债务风险。严格控制机构编制总量，财政供养人员只减不增。强化行政问责和监督。

关切民意诉求，畅通“民声通道”。继续开展“大排查、大走访、大接访”活动，进一步完善民情恳谈日、局长接待日制度，充分利用市长公开电话、读报读网等有效载体，掌握民情、化解矛盾、促进和谐。全面开展社会稳定风险评估。逐步完善人民调解、行政调解、司法调解联动工作体系，建立调处化解矛盾纠纷的综合机制。

整合信息平台，畅通“服务通道”。按照“人性化、网格化、信息化”的要求，改进社会治理方式。整合应急处置、公共服务、城市运行等领域信息资源，建设社会管理服务信息平台。继续开展幸福社区创建活动，40%以上社区办公用房面积达到1000平方米。深入实施“六五”普法规划。普遍建立法律顾问制度。加强政行风建设。

深入持久地开展“全国文明城市”创建活动，推动城市文明程度不断提升。推进军民融合深度发展，争创全国双拥模范城“八连冠”。

同时，我们还要继续做好国家安全、审计、工商、质监、统计、民族、宗教、外事、侨务、供销、气象、档案、保密、人防、地方志、红十字等各项工作。

各位代表，明年的任务艰巨而繁重，我们肩负的使命光荣而重大。

我们一定要转变工作作风，直面困难，敢于担当，真抓实干，雷厉风行，以朝气蓬勃、昂扬向上的精神状态狠抓落实、成就事业。

我们一定要坚持解放思想，以改革创新的精神统领各项工作，冲破思想观念的障碍，突破利益固化的藩篱，破除体制机制的弊端，让改革红利竞相迸发、充分涌流。

我们一定要密切联系群众，始终把人民利益摆在第一位，坚持不懈地为群众办实事、解难题、谋福祉，让发展振兴成果更多更公平地惠及广大人民群众。

我们一定要强化反腐倡廉，从源头预防腐败，规范行政权力运行，不断加强队伍建设，努力赢得人民的信赖支持，建设让人民满意放心的政府。

各位代表，新的一年马上就要开始了，我们即将踏上充满希望的新征程。让我们在市委的坚强领导下，万众一心，开拓奋进，共同创造长春更加美好的明天！
